# Resurgence of magnetic resonance techniques in the era of AlphaFold

**DOI:** 10.1007/s12551-025-01329-3

**Published:** 2025-07-18

**Authors:** Caitlin E. Skinner, Bethany A. Haynes, Rivka L. Isaacson

**Affiliations:** Department of Chemistry, King’s College London, Britannia House, 7 Trinity Street, London, SE1 1DB UK

**Keywords:** AlphaFold, Nuclear magnetic resonance, Electron paramagnetic resonance

## Abstract

Structural biology has seen the evolution of multiple pioneering experimental techniques over the last few decades, with leaps in technology and software facilitating rapid solution of crystal structures and the ‘resolution revolution’ in cryo-electron microscopy. Higher magnetic field strengths have expanded the development of magnetic resonance techniques and their ability to study protein dynamics and conformational diversity. Moreover, decades of experimental data collection and public data deposition combined with modern machine-learning technology have now made it possible to computationally predict three-dimensional protein structures from their amino acid sequence within minutes using AlphaFold (AF), a feat that has inspired a new wave of research. AlphaFold now contributes towards experimental structure solution and provides plausible predictions for structured regions of proteins leaving dynamics and conformational exchange as the next major questions in the field. Nuclear magnetic resonance (NMR) spectroscopy is uniquely placed both to rapidly validate AF predictions and probe protein dynamics at an atomic level in solution. Electron paramagnetic resonance (EPR) spectroscopy can measure distances between specific points in large protein complexes and provide local and global ranges of movement. This review will explore the revival of magnetic resonance techniques in a post-AlphaFold landscape and address their importance in protein research.

## Introduction to protein structure and dynamics

Proteins are polymeric biomolecules made up of linear chains of amino acids which fold into complex 3-dimensional structures. These shapes, which often entail folded domains connected by flexible linker regions, are responsible for the vast range of functions that proteins undertake within biological systems and convey specificity of interaction with protein and non-protein binding partners (Morris et al. 2021). Detailed structural analysis of proteins has been invaluable in developing our understanding of how their chemistry allows them to facilitate essential biological pathways. In 1960, X-ray diffraction of crystalline haemoglobin revealed the coordination of iron atoms by a heme structure that resides at the centre of each folded haemoglobin chain, elucidating how blood is able to carry oxygen through the body (Perutz, et al. 1960). Evolutionary relationships between proteins often demonstrate that common structural features may be conserved across proteins even in cases of low sequence similarity, suggesting that structure is more conserved than sequence (Chothia and Lesk 1986; Lee et al. 2007).

In a historic study of disulfide bonds within ribonuclease, Anfinsen concluded that the native protein structure is primarily determined by the linear sequence of amino acids (Anfinsen et al. 1961). Rapid Sanger sequencing of DNA (Sanger et al. 1977; McGinn and Gut 2013) has revolutionised the speed, volume and accuracy of the acquisition of gene, and by extrapolation protein, sequence information and has allowed the study of entire genomes of newly discovered microbes and viruses. In light of these developments, the field of bioinformatics has grown and fanned the flames of interest in the relationships between sequence, structure and function of biomolecules (Zhang and Wang 2023). Levinthal studied how proteins fold and concluded that there are specific pathways that occur with thermodynamic and kinetic control (Levinthal 1968). Molecular dynamics simulations have been employed to further investigate this process, leading to increased understanding of probable protein folds (Lindorff-Larsen et al. 2011).

Over the years, it has become increasingly clear that proteins are highly dynamic molecules, that have degrees of flexibility vital for their functions. Conformational changes in protein molecules are necessary for enzyme reactions, to enable the molecule to both bind the substrate and release the product, allosteric functions of proteins and enzymes, and the roles of proteins with contractile motor functions (Berendsen and Hayward 2000; Ishima and Torchia 2000). This phenomenon is seen in the HIV-1 protease enzyme, an aspartic acid protease which is required for HIV-1 maturation and infectivity (Nicholson et al. 1995). Early crystal structures of the homodimeric protein observed structures referred to as ‘flaps’ which adopted a closed conformation in the substrate bound enzyme (Miller et al. 1989). Additional NMR experiments further characterised the flaps as highly mobile and dynamic regions (Torbeev et al. 2011) that exhibited rapid changes in spatial conformation driven by isomerisation of conserved glycine residues (Ishima and Louis 2008), and that movement of these flaps was critical for catalytic activity of the enzyme (Torbeev et al. 2011).

As well as being important to their function, protein motion is necessary to facilitate folding from the emerging linear sequence, as it is polymerised from individual amino acids on the ribosome, into the final 3-dimensional structure (Berendsen and Hayward 2000). Fluctuations in energy and thermal motion can induce the formation of various final structures that each lie at energy minima, and proteins may transform between them (Berendsen and Hayward 2000). The dynamic properties and equilibrium between different structures provide further insight into function; however, this typically requires sophisticated computational methods to understand (Berendsen and Hayward 2000; Ishima and Torchia 2000). Many experimental and computational approaches have been employed to better our understanding of the complicated nature of proteins.

### Experimental approaches to structural determination

**Fig. 1:**
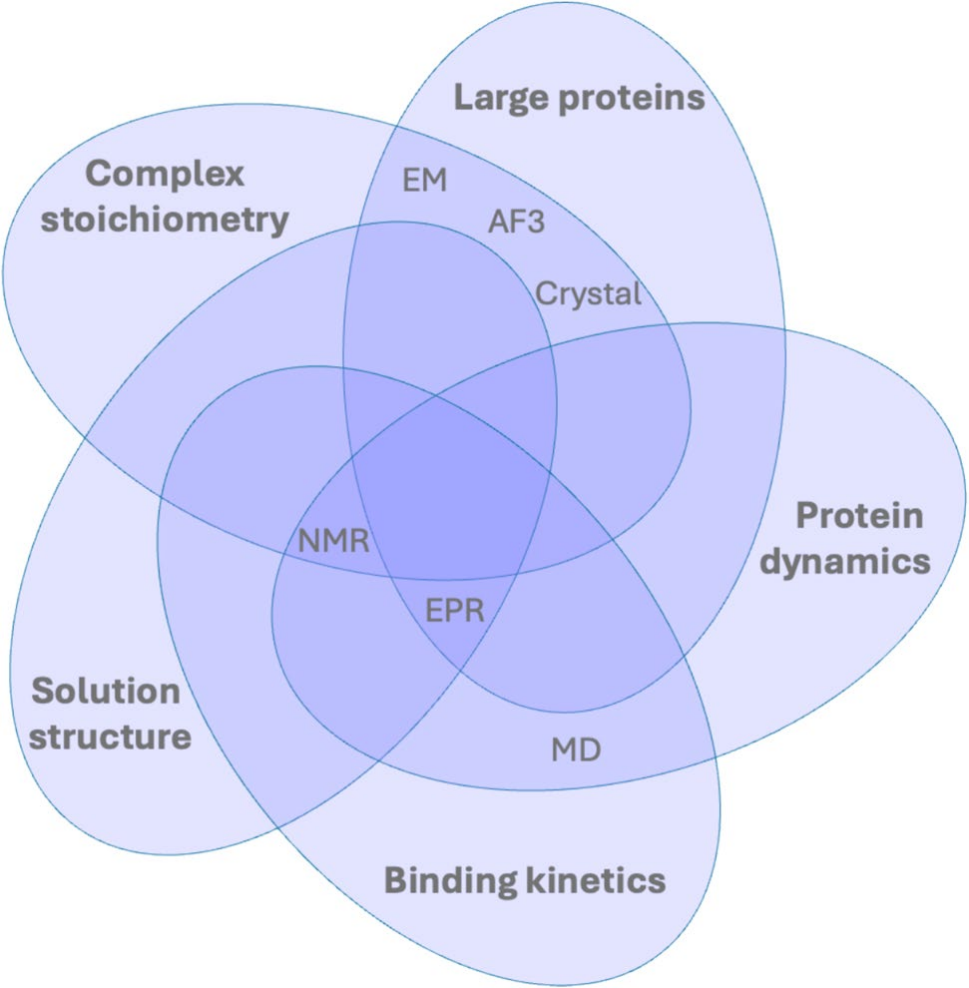
Venn diagram summarising key structural determination and prediction techniques and categorised by which aspect of protein structure and function they are suited to address. Electron microscopy (EM), AlphaFold 3 (AF3), X-ray crystallography (Crystal), nuclear magnetic resonance (NMR), electron paramagnetic resonance (EPR) and molecular dynamics (MD)

Experimental techniques for structure determination have shaped the structural biology field and led to extensive, publicly accessible databases, most notably the Protein Data Bank (PDB), of Ångström resolution macromolecular structures (Berman et al. 2007). For decades, X-ray crystallography, cryo-electron microscopy (cryo-EM) and magnetic resonance techniques have dominated the field, each bringing their own strengths and advantages. X-ray crystallography and cryo-EM can both provide static representations of proteins at near atomic resolution—in X-ray crystallography, conditions must be found in which the protein crystallises, and, for cryo-EM, the protein is preserved in a thin amorphous ice film (Venien-Bryan et al. 2017). X-ray crystallography examines the interaction between X-ray waves and electrons, such that they can be localised onto the asymmetrical crystal units to generate an electron density map from the diffraction data (Papageorgiou et al. 2021). This provides information on the atoms within the structure, their Cartesian coordinates, occupancy and displacement parameters, so that the structure can be resolved with atomic level resolution (Papageorgiou et al. 2021). For cryo-electron microscopy, low levels of radiation are used to produce micrographs that are used to generate a 3-dimensional model of the structure, using subtomogram averaging or single particle analysis methods (Guaita et al. 2022). Thanks to a step change in detector technology, structures that stay still enough can now be resolved at atomic resolution (Guaita et al. 2022). These methods provide reliable, experimentally validated, residue-specific structural information. Although more accessible than they once were, both techniques still face challenging sample preparations and require conditions that lack biological context (García-Nafría and Tate 2021). In many cases, a complement of techniques is best employed to address multiple aspects of structure and function (Fig. 1).

Whilst X-ray crystallography and cryo-EM mostly provide valuable snapshots of proteins in fixed conformations, magnetic resonance techniques have been able to address the pressing questions surrounding protein fluidity and the dynamics of biological systems (Henzler-Wildman and Kern 2007; Puthenveetil and Vinogradova 2019). Magnetic resonance techniques can be used to investigate the structure, dynamics and interactions of protein structures in solution (Sahu and Lorigan 2020; Bax and Clore 2019). As opposed to other biophysical techniques, the ability to investigate structure in solution facilitates a closer approximation to physiological conditions (Busch et al. 2025). This allows for the study of membrane proteins, disordered proteins, and conformational changes during biological processes (Ishima and Torchia 2000; Busch et al. 2025). Both nuclear magnetic resonance (NMR) and electron paramagnetic resonance (EPR) offer a range of experimental insights into protein structure and function.

Nuclear magnetic resonance spectroscopy uses a strong magnetic field to provide information on the spatial coordinates of nuclei which exhibit the property of ‘spin 1/2’. This includes naturally occurring ^1^H and rarer isotopes ^13^C and ^15^N which are introduced into proteins by scientists (Bax and Clore 2019). The local chemical environment of these nuclei within protein structures gives them unique and distinguishable behaviours within the spectrometer. As well as enabling protein structure determination, dynamic properties can be studied on a huge range of timescales, including picosecond side chain rotation, nanoscale molecular tumbling, folding, catalytic activity and domain motion at slower timescales (Ishima and Torchia 2000).

Protein structural analysis by NMR relies upon distance restraints obtained from specific NMR experiments, primarily looking at nuclear Overhauser effects (NOE), J-coupling and residual dipolar coupling (RDC) (Bax and Clore 2019). NOEs show how excited nuclei returning to equilibrium are affected by nearby coupled spins, as a function of distance; this allows protein NOESY spectroscopy to determine internuclear distances through space (Bax and Clore 2019) whilst J-coupling effects occur through bonds between neighbouring atoms.

RDC measurements require an alignment medium and examine the rotational average of the magnetic dipole–dipole interaction between nuclei that deviate from zero under weak anisotropic conditions. They can be used to elucidate information on bond orientations within structures (Bax and Clore 2019). Another powerful kind of NMR analysis, paramagnetic relaxation enhancement (PRE) is possible when proteins contain unpaired electrons, e.g., paramagnetic metal ions (Ravera et al. 2022). These can occur naturally or be introduced as probes to determine specific parameters such as the orientation of a peptide in a binding site (Thapaliya et al. 2016). Paramagnetism in NMR is a source of information on both biomolecular structure and dynamics, with PRE measuring the effect of dipolar interactions between nuclei and unpaired electrons on magnetisation relaxation, also as a function of distance. This can provide long-range distance restraints of proteins that are less susceptible to internal motions and cross-relaxation, to be applied for structural refinement and validation (Busch et al. 2025). Multidimensional NMR spectra such as these provide the volume of information required to study the structures of macromolecules, including proteins (Bax and Clore 2019). Assignment strategies relate cross peaks of the multidimensional spectra to specific nuclei allowing for the proper positional interpretation of the NOE, PRE and RDC information (Bax and Clore 2019). Measures of protein dynamics and flexibility can be determined from NMR according to the exchange rates of protons, spin–lattice relaxation rates and spin–spin relaxation rates (to determine the rotational diffusion tensor), and the bond order parameters (Ishima and Torchia 2000). The information obtained by NMR provides insight into the structure at an atomic level, with considerations for the conformational entropy and non-covalent interactions that are likely to be involved in weak, transient and dynamic interactions (Busch et al. 2025).

The collection and analysis of such comprehensive and informative data requires sophisticated technology and significant time and effort from researchers which can be daunting. However, when used to validate AlphaFold predictions and augment them with information on dynamics, binding and changing conformations, these experiments become more targeted and less laborious in a true win–win situation (Laurents 2022). This review will discuss the benefits of combining structure prediction software AlphaFold with magnetic resonance techniques and present a selection of case studies showcasing the synergistic potential.

### AlphaFold

The development of artificial intelligence (AI) and machine-learning techniques has provided a turning point within protein structure prediction methods. Namely, Google DeepMind’s development of AlphaFold, a programme that generates predicted protein structures from amino acid sequences, was awarded the 2024 Nobel Prize in Chemistry for its impact (Tunyasuvunakool et al. 2021; Jumper et al. 2021a). The AlphaFold Protein Structure Database (Varadi et al. 2022) offers immediate free access to predicted structures for almost every known protein, whilst the AlphaFold server (Jumper et al. 2021a) allows anyone to predict the structure of a protein or complex of interest, often within minutes. Hence, atomic-level protein structure predictions are now readily accessible to all, and it has rapidly become commonplace to see protein structures in talks by non-structural biologists.

AlphaFold predicts structure according to molecular driving forces and evolutionary protein history—experimentally determined structures publicly deposited within the Protein Data Bank (Berman et al. 2007) (PDB) were used as training data and the evolutionary, physical and geometric constraints were used to generate the neural network architecture (Jumper et al. 2021a). From this, the 3-dimensional coordinates of the heavy atoms within the sequence are predicted with a report of the expected per residue accuracy (indicative of the confidence) and a predicted local-distance difference test (pLDDT) (Jumper et al. 2021a). Unlike prior prediction methods, AlphaFold is able to provide a possible structure without specific template structures or high degrees of sequence similarity (Tunyasuvunakool et al. 2021). This is useful for the study of proteins that were underrepresented in the training data (Tunyasuvunakool et al. 2021).

The impact of AlphaFold came to the fore in the Critical Assessment of Structure Prediction (CASP), a community experiment run every 2 years to monitor the accuracy of arising methods within the field by comparing data gathered by theoreticians and experimentalists (Moult et al. 2014). The introduction of AlphaFold into this experiment in 2020 substantially increased the average success in generating accurate predictions (Jumper et al. 2021b). In CASP13, it was regarded as a notable advance to have the performance trendline exceed a global distance test score (GDT_TS) of 60; in CASP14, with the introduction of AlphaFold, the GDT_TS was lowest at 85 for the most difficult targets (Jumper et al. 2021b). Analysis of CASP14 data showed that AlphaFold-based models were pivotal for the improvement in performance, relative to CASP13 (Kryshtafovych et al. 2021). In addition to the impact of AlphaFold on performance, it is important to note the impact of AlphaFold on the organisation of CASP experiments. For each yearly submission, there is a selection of categories for focus. Prior to AlphaFold, these categories looked at more general areas of analysis, for example 3-dimensional contacts, refinement and estimations of accuracy (Kryshtafovych et al. 2021). In the wake of AlphaFold, these could largely be regarded as solved, and the categories in CASP15 focused on specific areas of study, for example RNA structures and protein-small ligand complexes (Kryshtafovych et al. 2023).

**Fig. 2:**
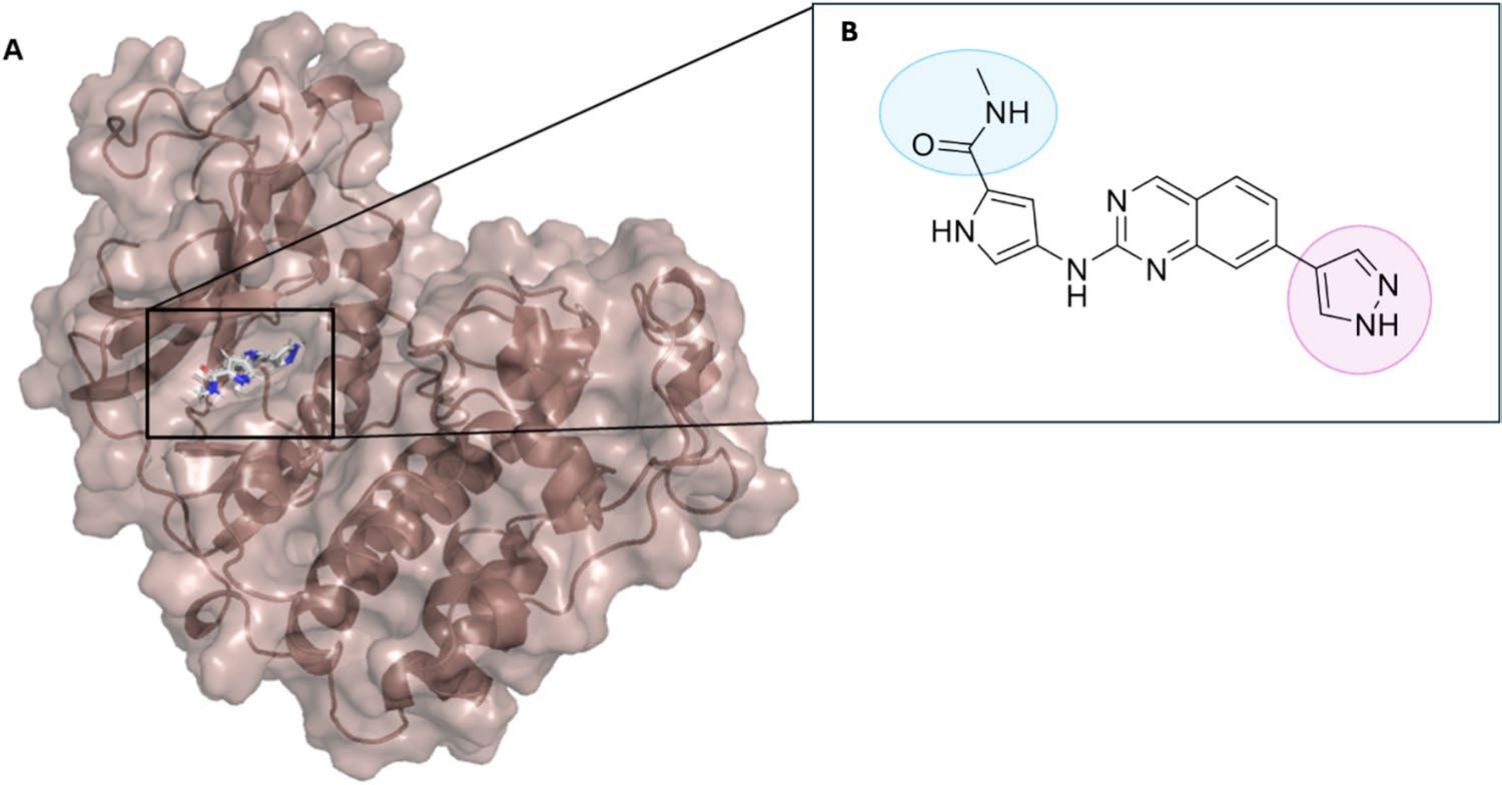
**A.** An AlphaFold predicted structure of CDK20 (1–302) with small-molecule inhibitor docked using SwissDock (Bugnon et al. 2024). **B.** Chemical structure of small molecule CDK20 inhibitor ISM042-2–048 highlighting cis–trans isomerisation of the pyrrole-2-carboxamide group (blue) and the addition of a diazole to the quinazole group (pink). Figure was created using data from Ren et al. (2023)

Developments with AlphaFold have prompted applications within structure-based drug discovery (Ren et al. 2023; Baselious et al. 2023). A study was conducted to develop small-molecule inhibitors for CDK20, a kinase protein involved in the cell cycle that represented a target for cancer treatment (Ren et al. 2023). AlphaFold was employed to generate a model of the CDK20 ATP-binding pocket (residues 1–302) (Fig. 2A) as an input for AI tool Chemistry42 to design small-molecule inhibitors (Ren et al. 2023). Essential residues and dimensions of the CDK20 binding pocket were used to influence compound design by functionalisation of a quinazole ring and chemical modification of a pyrrole-2-carboxamide group (Fig. 2B), facilitating access to the solvation region via interaction with key acidic residues (Ren et al. 2023). These design features generated a compound that when synthesised and tested showed a binding affinity with a Kd value of 566.7 ± 256.2 nM and demonstrated anti-proliferative activity in cells (IC_50_ = 208.7 ± 3.3 nM) compared to a control (IC_50_ = 1706.7 ± 670.0 nM) (Ren et al. 2023).

However, the use of AlphaFold in drug development initially faced difficulties due to the absence of cofactors and small molecule ligands in the generated structure (Baselious et al. 2023). This presented issues with the development of histone deacetylase 11 (HDAC11) inhibitors (Baselious et al. 2023). The AlphaFold predicted structure was deemed reliable with respect to the core folds and the catalytic core aligned with what would be expected from related deacetylases; however, the positioning of crucial flexible regions about the active site had low confidence, and the zinc cofactor binding could not be predicted. This led to an inability to appropriately predict possible inhibitors, as, relative to experimentally determined structures, existing inhibitors could not be correctly positioned (Baselious et al. 2023). Challenges associated with structural predictions involving small ligands are expected to improve with the release of AlphaFold3, the newest version which permits the inclusion of certain metals and cofactors (Abramson et al. 2024), and such subtleties are an important area in which NMR can help validate predicted distances.

## AlphaFold and experimental methods: an integrative approach

Whilst AlphaFold represents a huge step-change in structure prediction and accessibility, there are still many unanswered questions and areas for improvement. Membrane proteins still pose a problem for AlphaFold (He et al. 2025), as do proteins with multiple possible conformations, where AlphaFold usually zones in on the lowest energy option with no way of accessing the others (Chakravarty et al. 2024). AlphaFold also sometimes overpredicts rigidity, a function of the PDB training set having been dominated by crystal structures (Agarwal and McShan 2024). AlphaFold offers prediction rather than experimental evidence, and slowly some cases are emerging in which a high-confidence prediction from AlphaFold disagrees with experimental data (Bonin et al. 2024; Massoni et al. 2025). It is therefore vital that in cases where atomic detail is required, prediction must be backed up by experimental measurements, and both NMR and EPR, with their abilities to determine through-space distances between atoms, are invaluable for this purpose.

Moreover, integrated use of AlphaFold with experimental structure determination methods has shown great promise as a more efficient means of accurate structure resolution. To solve the ‘phase problem’ in X-ray crystallography, molecular replacement can often be used to reconstruct the electron density map (Wang et al. 2025) (22). However, in instances where a similar structure was not available, this was not previously possible and required alternative laborious solutions such as heavy atom replacement (Crichton and Louro 2013). Now, AlphaFold can be used to provide molecular replacement in the form of a relevant predicted structure (Wang et al. 2025). During the study of the bacterial protein Q63NT7 with X-ray crystallography, there was missing phase information because of the size of the complex, and flexibility of the C-terminal region led to difficulty forming an appropriate crystal (Miller et al. 2024). Information from the predicted AlphaFold structure was used to complement the experimental information and solve the crystal structure (Miller et al. 2024).

Flexibility within molecules can also be problematic for structure resolution by other techniques including X-ray Free Electron Laser (XFEL) spectroscopy and cryoEM (Noone et al. 2022). AlphaFold was used to resolve a mobile region in PTX3, a pattern recognition molecule being studied for targeting agents due to its role in COVID-19 prognosis (Scavello et al. 2024), cancer progression (Bogdan, et al. 2022), and female infertility (Camaioni et al. 2018). A cryo-electron map was generated for the C-terminal domains and found to form an octameric complex with disulphide bonds and surface glycosylation; however, mobility of the N-terminal domain led to a lower resolution map and required the use of AlphaFold to confirm the structure (Noone et al. 2022). Congruence was found between the AlphaFold model and the octameric complex generated with cryo-EM, and the generated structure had 4 identifiable binding sites that present possible targets for drug development (Noone et al. 2022).

The chemical shift assignment for NMR studies is crucial for extracting the desired insight from the spectra, studying residue-level binding and dynamics, and solving three-dimensional structures; however, this process makes NMR protein studies very labour-intensive (Bax and Clore 2019). It is in this way that machine-learning programmes, such as AlphaFold, can be employed to accelerate and complement NMR-based studies (Klukowski et al. 2023; Alderson et al. 2023; Huang et al. 2024). Research has found that different collaborations of multidimensional NMR spectroscopy work complementarily with AI programmes to minimise the measurement time and automate chemical shift assignments (Klukowski et al. 2023). The use of AlphaFold to inform chemical shift assignment was found to yield an average backbone RMSD of 1.03 Å, relative to experimentally determined structures within the Biological Magnetic Resonance Data Bank (BMRB), and using AlphaFold to inform UCBShift predictions gave an average backbone RMSD of 0.85 ppm (Klukowski et al. 2023). This indicates that, within a margin of error, these programmes can be used to estimate assignments from NMR-based studies. Also, with AlphaFold generating an ensemble of structures of varying confidence, multiple candidates can be considered as an analogy of an NMR bundle for more accurate predictions (Klukowski et al. 2023). The use of AlphaFold and UCBShift to inform predictions made by ARTINA (an AI programme for automated chemical shift assignments) provided an accuracy of 95.45% with the use of 5 3-dimensional NMR spectra (^15^N-edited [^1^H,^1^H]-NOESY, ^13^C-edited [^1^H,^1^H]-NOESY, CBCAcoNH, HCCH-TOCSY and CCH-TOCSY), relative to 91.37% without the use of AlphaFold structures (Klukowski et al. 2023). This suggests promise for improving the ability of auto-assignment programmes to minimise the labour required for NMR studies of proteins.

Another argument for the integrated use of AlphaFold with NMR is that AlphaFold often cannot accurately portray the dynamics of regions which exhibit flexibility (Stevens and He 2022). In an accuracy of NMR structures using RCI (Random Coil Index) and rigidity (ANSURR) assessment to probe the fidelity of selected dynamic structures, NMR solution structures outperformed the predicted structures generated by AlphaFold (Fowler and Williamson 2022). Conversely, and judged against the same parameters, in some cases, analysis found increased accuracy of AlphaFold structures relative to NMR structures (Fowler and Williamson 2022). This indicates that the use of a collaborative approach would be able to broadly cover structural resolution and account for the errors of the individual methods themselves, some examples of which are presented below.

Mucosa-associated lymphoid tissue lymphoma-translocation protein 1 (MALT1) is of interest in medicinal chemistry as a target for cancer treatment due to its role in regulating adaptive immune response pathways following antigen stimulation (Wallerstein et al. 2024). During signalling, MALT1 may behave as both a scaffold and a protease (Jaworski and Thome 2016). The dual activity of MALT1 is believed to be regulated by allosteric modulation at an interface between the C-terminal PCASP and Ig3 domains (Pelzer et al. 2013). The structure of these domains has previously been determined by X-ray crystallography (Wiesmann et al. 2012); however, further studies were required to address the changes in protein motion, triggered by allosteric regulators, that facilitate the duality of MALT1 (Wallerstein et al. 2024). A study employed NMR ^15^N relaxation and methyl-NOE experiments to identify regions of fast exchange between the PCASP and Ig3 domains, suggesting the presence of highly dynamic regions and conformation heterogeneity (Wallerstein et al. 2024). The identified regions were compared against AlphaFold predictions of the same construct, where low pLDDT scores were found to coincide with the experimentally determined dynamic regions. Further principal component analysis of the generated AlphaFold models identified prominent conformational ensembles, which directed attention to an essential tryptophan (W580) that was found to be pivotal in the allosteric changes which control protein activity (Wallerstein et al. 2024). This application of an integrated AlphaFold-NMR approach has promise for the study of inhibitors, which otherwise use 2D molecular interaction analysis, due to ambiguity surrounding the conformation of the binding site (Alshehri et al. 2024).

Electron paramagnetic resonance (EPR) spectroscopy utilises similar principles to NMR but, rather, measures the absorbance of microwave radiation by unpaired electrons on paramagnetic species (Sahu and Lorigan 2020). Within biological macromolecules, paramagnetic species may be in the form of metal cofactors, reactive oxygen species or labels on the molecules, using site-directed spin labelling (SDSL) methods (Torricella et al. 2021). Labels used in EPR must have selective reactivity and appropriate sensitivity, whilst avoiding disruption to the structure (Goldfarb 2022). The dipolar coupling between paramagnetic labels can be measured and used to find the distances between atoms (as they are inversely proportional) (Goldfarb 2022). This can be used to extrapolate information regarding the structure, such as long-range restraints, conformational changes and dynamic information (Goldfarb 2022). EPR can be conducted using continuous wave or pulse methodology depending on the molecular information sought (Torricella et al. 2021). Double electron–electron resonance (DEER) EPR is a pulse-EPR spectroscopy that is typically used to measure distances between paramagnetic probes installed on proteins and interrogate their potential for dynamic motion, structure, and interaction sites (Sahu and Lorigan 2020; Torricella et al. 2021).

EPR has been used in conjunction with AlphaFold structures to study the CCR4-NOT complex, which is involved in regulatory mRNA degradation (Zhang et al. 2022). X-ray crystallography of a related complex had generated a structure (CHen et al. 2021); however, at one of the catalytic sites, there was poor resolution, so AlphaFold was used as a molecular replacement. The finalised structure was then used as a scaffold to study the metal ion cofactors at the catalytic sites (Zhang et al. 2022). Pulse-EPR was then utilised as a confirmatory technique of the proposed model. A SDSL technique was used such that paramagnetic metal ions (Mn^2+^) occupied the sites where cofactors bind in the native form, such that information pertaining to their binding could be derived (Zhang et al. 2022). This displays an application of EPR spectroscopy in an integrated AlphaFold approach to improve the quality of structural models developed to include important binding factors, which has previously been an issue with AlphaFold, although less so now with the advent of AlphaFold3 (Abramson et al. 2024).

Another EPR-AlphaFold integrated approach was adopted for the study of the amino acid polyamine organocation (APC) transporter, GadC (Alamo et al. 2022). This transporter is involved in the exchange of extracellular glutamate and intracellular γ-aminobutyric acid (GABA) to facilitate the survival of pathogenic bacteria in acidic conditions (Kanjee and Houry 2013). X-ray crystallography and cryo-EM have resolved structural elements of the GadC protein and human orthologs (Yan et al. 2019); however, the conformational dynamics had not been resolved such that a mechanism for transport could be determined (Alamo et al. 2022). AlphaFold was used to generate a comprehensive set of conformationally diverse ensembles of the GadC transporter. These conformations were used to direct DEER-EPR experiments designed to monitor the conformational dynamics under different conditions of pH and substrate concentration (Alamo et al. 2022). The changes in distance between spin labels induced by pH-driven shifts in conformational equilibrium were in agreement with predictions made by AlphaFold, demonstrating the ability of AlphaFold to streamline spectroscopic approaches (Alamo et al. 2022).

## Conclusion

Whilst AlphaFold provides efficiencies in the use of traditional experimental protein structure solution methods, it can also offer a plausible alternative to them in cases where its predictions come with high confidence. The ready availability of predicted structures in the AlphaFold database (Varadi et al. 2022), and the opportunity for anyone to rapidly predict their own structures using the AlphaFold server (Abramson et al. 2024), has democratised molecular structure and led to interest from many research areas that previously found it inaccessible. AlphaFold has moved us on to the next frontiers in understanding protein structure, those of dynamics and conformational variability, particularly in larger multi-protein systems. Magnetic resonance techniques will prove to be invaluable partners in these future quests, and their collaboration with AlphaFold is really just beginning.
